# They live under our streets: ant nests (Hymenoptera, Formicidae) in urban pavements

**DOI:** 10.3897/BDJ.11.e102897

**Published:** 2023-04-27

**Authors:** Louise Dijon, Wouter Dekoninck, Gilles Colinet, Frédéric Francis, Grégoire Noel

**Affiliations:** 1 Functional and Evolutionary Entomology, Gembloux Agro-Bio Tech – University of Liège, TERRA, Gembloux, Belgium Functional and Evolutionary Entomology, Gembloux Agro-Bio Tech – University of Liège, TERRA Gembloux Belgium; 2 Royal Belgian Institute of Natural Sciences, Brussels, Belgium Royal Belgian Institute of Natural Sciences Brussels Belgium; 3 Soil-Water Plant Exchanges, University of Liège, Gembloux Agro-Bio Tech, Gembloux, Belgium Soil-Water Plant Exchanges, University of Liège, Gembloux Agro-Bio Tech Gembloux Belgium

**Keywords:** urban ecology, urban conservation, ground-dwelling ant, *Lasiusniger*, urban construction

## Abstract

In the context of global insect decline, the urbanisation process plays a key role. However, urban pavements, which are considered to be impervious to biodiversity, can harbour ground-nesting insects under certain conditions. Recent observations have revealed the presence of Formicidae nests under urban pavements. The aim of this work is to determine the species richness of Formicidae nesting under urban pavements in the Brussels-Capital Region (Belgium) and to characterise their nest environment and soil texture. Seven ant species were identified in 120 nesting sites: *Lasiusniger*, *Lasiusbrunneus*, *Lasiusflavus*, *Lasiusfuliginosus*, *Tetramoriumcaespitum*, *Tetramoriumimpurum* and *Myrmicarugulosa*. Concrete slabs or natural stones with a sandy sub-layer are the main structures in which ants nest. In addition, nests were mainly found under modular pavements with degraded rigid joints. The results of this work highlight the capacity of urban structures to host part of ant biodiversity in cities.

## Introduction

Since the last century, human populations have shown an increasing interest in urban life, leading to the expansion of cities and the number of their inhabitants (Grimm et al. 2008). The urbanisation process, or the conversion from natural areas to anthropogenic areas, is responsible for the fragmentation, isolation and degradation of surrounding habitats (Clarke et al. 2008). These disturbances reshape species assemblages and lead to the formation of living communities adapted to urban ecosystems (Yamaguchi 2004, McDonald et al. 2019). Soil habitats are of fundamental importance for some species, especially ground-nesting insects (e.g. in Antoine and Forrest (2021)). Recent studies focusing on the insect biodiversity of urban areas revealed the presence of several Hymenoptera species which can nest under urban pavements. These studies focused mainly on the nesting ecology of solitary bees and digger wasps, but also recorded some ant species (Noël et al. 2021, Weber 2022).

Ants play an important role, mainly as decomposers, in many ecosystems around the world. The biomass of ground-dwelling ants exceeds the combined biomass of wild birds and mammals and represents the fifth of the human biomass (Schultheiss et al. 2022). Moreover, ants play an essential role in soil structure and energy flow; hence, some consider them as the engineers of ecosystems (Folgarait 1998). Nutritional needs of each species, nest density, species richness and community structure can vary greatly depending on habitat space and available food resources (Wegnez et al. 2012, Feldhaar 2014). Site selection is crucial for colony development and several biotic and abiotic factors appear to influence its density and distribution. Most ant colonies nest in the soil and are sensitive to criteria, such as grain size composition and moisture that influence the burrowing, stability and development of their colony (Diaz 1991, Kaspari et al. 2000, Wang et al. 2001, Boulton et al. 2005, Clarke et al. 2008). The surrounding vegetation also plays a significant role to ant communities. The richness, abundance and size of vegetation patches influence ant species richness (Boulton et al. 2005, Uno et al. 2010, Cerdá et al. 2013). Related to the presence of vegetation, another criterion that influences the location of a colony is the availability of food resources near the nest. The range of movement varies with species, colony density and food availability. This radius can vary from 50 cm to sometimes 200 m (Carroll and Janzen 1973, Savolainen et al. 1988). Finally, foraging activity is particularly influenced by climatic variations (Cros et al. 1997). Temperature seems to be the first criterion that regulates the activity of ants. The optimal temperatures for ant activity are between 10°C and 45°C (Hölldobler and Wilson 1990), but some tropical species can increase their critical thermal maximum (i.e. the maximum temeprature at which an organism is unable to resist heat stress) over 50-55°C (Leong et al. 2022) which is also associated with the diminution of their foraging activity (Cerdá et al. 1998).

In general, studies relating to the ecology and diversity of ants in urban environments are mainly concentrated in parks and green spaces (Yamaguchi 2004, Pacheco and Vasconcelos 2007, Uno et al. 2010, Ślipinski et al. 2012). These studies highlight a negative relationship between species richness and proximity to urban development (Sanford et al. 2009), such as green areas bordering streets (Ślipinski et al. 2012) or in isolated park areas (Santos et al. 2019), but this pattern is not always observed in every study. Idiosyncrasies of other assessed cities may favour or maintain ant species richness in urban areas (Guénard et al. 2015, Miguelena and Baker 2019, Perez and Diamond 2019). Ants are an integral part of urban ecosystems, but their ecology within urban constructions is still rather poorly documented. Here, we hypothesised that urban areas present thermophilic zones particularly attractive for ants. Hence, urban pavements could constitute a suitable thermal refuge for their nest under certain conditions, such as sufficient food resources, permeable pavements and favourable microclimatic conditions, such as high temperatures and relatively high humidity.

The aims of this study are to determine and locate the specific richness of ant colonies nesting under urban pavements in the city of Brussels-Capital Region (BCR; Belgium) and to characterise the main factors that could influence their nesting site selection.

## Materials and methods

### Study site

The study focused on urban pavements throughout the municipalities of BCR. The surface area of this region is 161.38 km^2^ and pavements represent 3600 km in length (Bruxelles Mobilité 2022). Fifty-four percent of the surface of BCR is non-built which corresponds to green spaces, such as forests, private gardens or parks (Van de Voorde et al. 2010). There is a contrast between the highly urbanised city centre and the periphery, which is like a green belt. The main green areas are in the east and southeast of Brussels (Bruxelles Environnement 2013). The remaining 46% are impervious surfaces (built-up), concentrated more towards the city centre (Bruxelles Environnement 2015). The sampling period took place in the spring from 12 April 2022 to 17 June 2022. BCR is characterised by temperate oceanic climate, the 10°C ambient temperatures necessary for ant activity being reached from April until October (Cerdá et al. 2013, Bruxelles Environnement 2020).

### Sampling methods and specimen preparation

**Figure 1.**
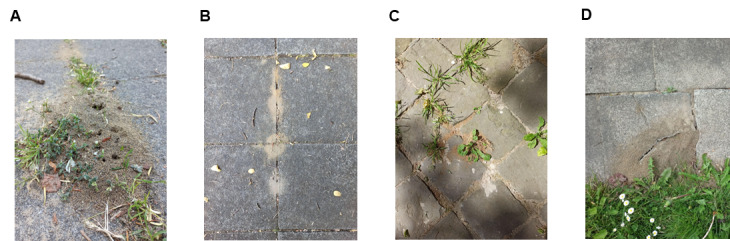
Ant nest on pavements. Sandy mound (Uccle, Brussels) (A). Degraded rigid joints of concrete slabs (Auderghem, Brussels) (B). Unbound joints of natural stone (Brussels) (C). Crack in a concrete slab (Berchem-Sainte-Agathe, Brussels) (D).

As a first exploratory study on the diversity of Formicidae nesting under the pavements of BCR, data were collected opportunistically. This sampling method allows for a larger geographical scope due to the time saved at each site and will give a better chance to find rare species. The locations from the dataset of Noël et al. (2021) were used as a basis for the search for nests in order to cover as much territory as possible with a high probability of observing ants. An anthill was located on a pavement, either thanks to the sand mound between two paving stones or thanks to an ant followed to its anthill (Fig. 1A). The site was then validated by detecting the activity of several workers outside the nest. Each site assessment was conducted for 10-20 min. Fieldwork was performed on sunny days with clear sky and a daily minimum temperature of 10°C between 09:00 and 17:00 h. Using a brush previously soaked in 70% alcohol (Delabie 2001), about ten specimens were collected for identification in the laboratory. All the specimens were identified using a stereomicroscope with taxonomic keys of Seifert (1996), Wegnez et al. (2012), Blatrix et al. (2013), and Seifert (2018). The identified specimens were cross-checked by reference collections of the Royal Belgian Institute of Natural Sciences (RBINS, Belgium) and vouchered to Gembloux Agro-Bio Tech (University of Liège, Belgium) and RBINS insect collections.

### Pavement characterisation

The width of the pavement (i.e. between the road and the constructed building) was measured at each site. Then, the joint width was measured at the nest exit with a ruler placed perpendicular to the joint length. The nature of the joint was encoded as a qualitative variable: rigid joint (Fig. 1B) / flexible joint (Fig. 1C) / other (Fig. 1D). The nature of the pavement was also considered as a nominal qualitative variable: asphalt / natural stone (Fig. 1C) / concrete slab (Fig. 1B). Finally, the location of the nest in relation to the pavement was also encoded: middle of the pavement / wall border / green border / street border.

### Granulometry

The soil excavated by the ants was collected at each site. It was not possible to extract the substrate below the paving stones due to the logistic and administrative costs of this practice. Consequently, a maximum of substrate taken out by the ants was collected. If several mounds were present, the substrate was collected from all mounds. Substrate samples were used to perform a particle size analysis. The soil texture definitions can vary according to the spatial scale of the studied geographic areas and the nature of the particles (Udden 1914, Wentworth 1922). We used the Bonnot-Courtois and Fournier's granulometric definitions in Fournier et al. (2012). Each sample was weighed (1/100 accuracy) and then passed through a sieve (Haver and Boecker VWR) for 5 minutes at an oscillation amplitude of 1 mm. Four sieves of mesh size 1 mm, 0.5 mm, 0.2 mm and 0.1 mm were used. It allowed us to differentiate coarse elements, coarse sands, medium sands, fine sands and very fine sands including clays and silts (Fournier et al. 2012). Rejects from each sieve were weighed and converted to relative mass to the total sample mass to allow for comparisons between sites (Fournier et al. 2012).

### Mapping and statistical analyses

Map analyses were performed using QGIS software - Version 3.18.1 (QGIS 2022). We retrieved a map raster of high and low vegetation in Brussels from Bruxelles Environnement (2022).

Statistical analyses and graphs were performed with R software v. 4.1.3 (RStudio Team 2022) and the following packages: *ggplot2* (Wickham 2016) and *corrplot* (Wei and Simko 2021). To estimate the true diversity of our sampling, we performed Chao1 (abundance-based) estimator of richness using the R package *iNEXT* (Hsieh et al. 2016)

After noting the dominant presence of the species *Lasiusniger* (Linnaeus, 1758), the statistical analyses focused on the comparison of this species compared to all the other species according to the particle size fractions. Indeed, the abundance of the latter was too low to study them individually. These comparisons were made using Student’s t test, Wilcoxon-Mann-Whitney test and Welch’s t test, depending on the normal distribution of the data and the homogeneity of variances.

## Results

### Ant diversity

**Figure 2.**
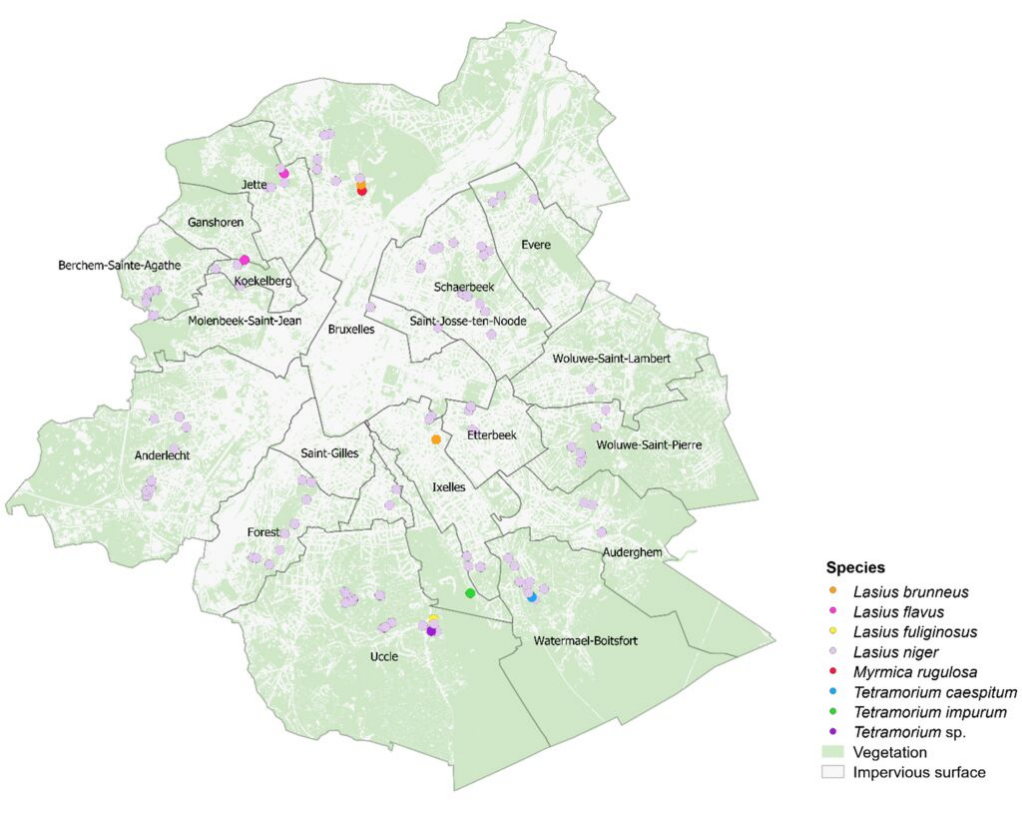
Ant nest location (coloured dots) in Brussels-Capital Region (Belgium). Green shade corresponds to the vegetation cover and white shade corresponds to the impervious surface.

In 17 municipalities of BCR, 120 sites with active ants were sampled (Fig. 2). Seven taxa were identified. These are grouped in two different subfamilies: Formicinae (4) and Myrmicinae (3). Regarding the abundance of these species, *L.niger* is dominant, occupying 92% of the sites. The six other species are present at a rate of 1 to 2% each (Table 1). The species identification process was not possible for two specimens of the genus *Tetramorium* which could be *Tetramoriumcaespitum* (Linnaeus, 1758) or *T.impurum* (Foerster, 1850). Hence, we classified these two specimens in the *Tetramorium* sp. complex.

**Figure 3.**
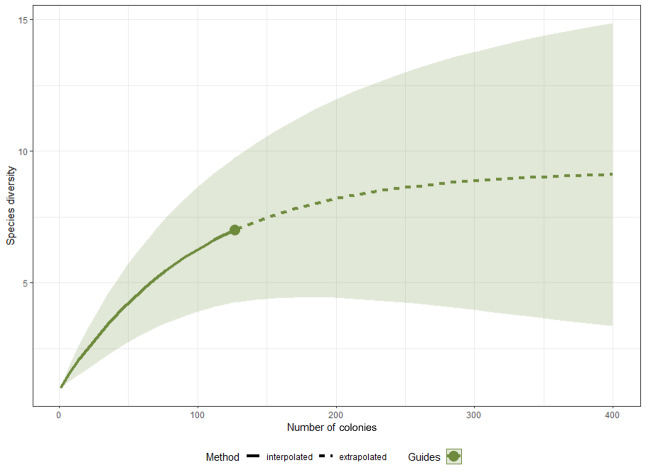
Rarefaction curve and its extrapolation from the 120 sampled sites. The green shade corresponds to the 95% interval confidence.

The rarefaction curve and the Chao1 index estimated that the expected species richness of nesting ants in pavements for BCR is around nine species with 95% of confidence interval between 7.26 and 25.91 species (Fig. 3).

### Pavement characterisation

**Figure 4.**
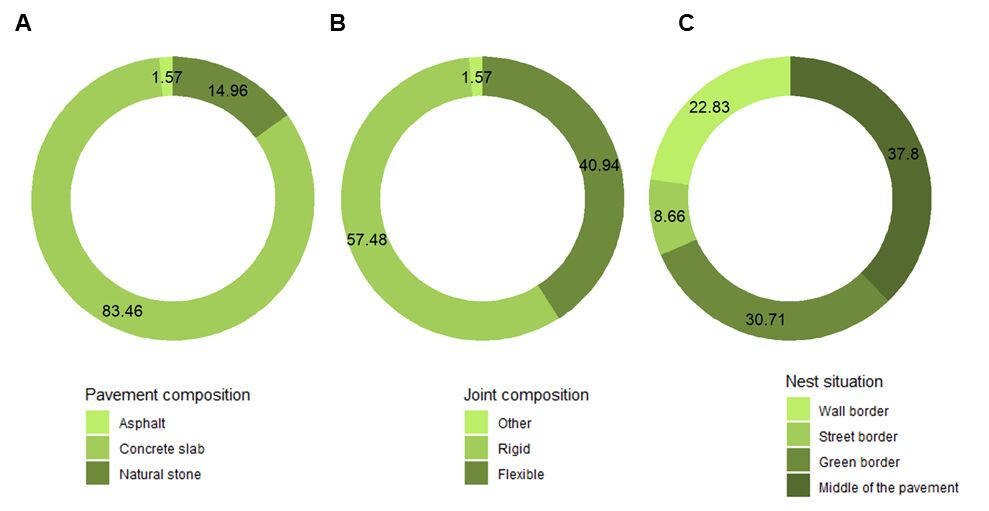
Pavement ring charts for the 120 sampled sites for pavement composition (A), joint composition (B) and nest location (C).

The dominant type of pavement was concrete slabs (83.5%). Nests were also found under natural stone pavement (15%) and at low frequency under asphalt (1.6%) (Fig. 4A). For the nature of the joints, 56.7% are rigid and 41.7% are flexible. The remaining 1.6% represent the other category which includes cracks in the asphalt (Fig. 4B). Finally, the position of the nests was classified into four categories. The middle of the pavement was the most represented with 37.8%. Some 30.7% were located at the border of green spaces, 22.8% at the border of a wall and a smaller proportion was located at the border of the street (8.7%) (Fig. 4C).

### Granulometry

**Figure 5.**
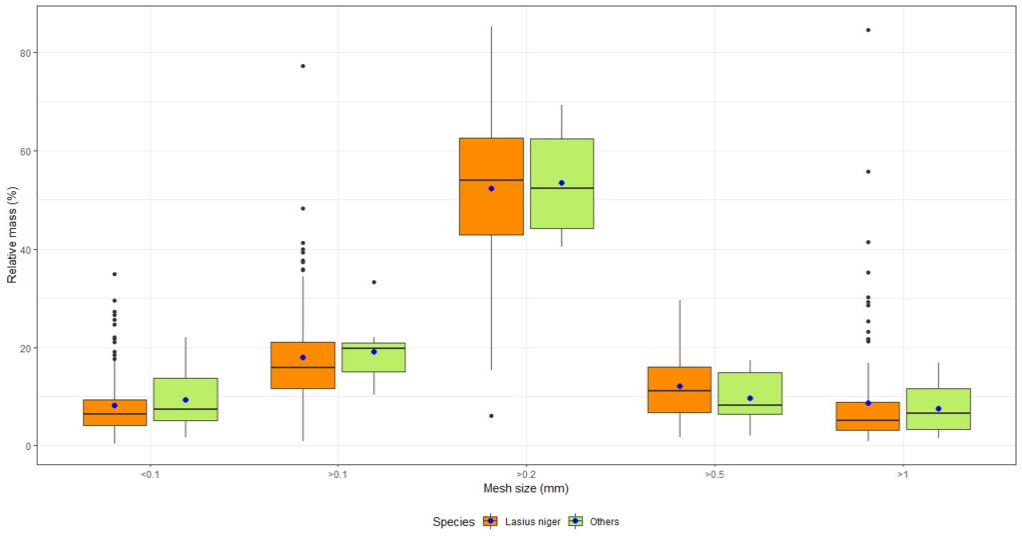
Boxplot per grain size class for *Lasiusniger* (orange) and other ant species (green). The blue dot corresponds to the boxplot mean of relative mass (%).

For the granulometric variables, the distribution of data is relatively similar between the two groups. There is no significant difference between *L.niger* and other ant species. The grain size class > 0.2 mm is present in the majority of samples with an average of more than 50% of the total mass. In addition, the class < 0.1 mm, which includes clays and silts, is less than 10% of the total mass of the sample (Fig. 5).

## Discussion

### Ant diversity

The ecology of ants in BCR pavements has been studied for the first time. Other studies have reported their presence in Berlin and Oldenburg in Germany (Haeseler 1982, Weber 2022). Some species are common to all three cities, such as *L.flavus, L.niger* and *T.caespitum.* Others were identified for the first time in our study, such as *L.brunneus, L.fuliginosus, M.rugulosa* and *T.impurum.* Our study showed a similar species richness to the Weber (2022) study in Berlin with seven different species, whereas the Haeseler (1982) study in Oldenburg found only two species. These differences in richness can be explained by the different sampling methods and effort between the studies. In BCR, a larger area was surveyed in Brussels than in the other two studies, which focused on predetermined smaller sites (Haeseler 1982, Weber 2022). Moreover, the 120 new ant records complete the Formidabel database, which includes 535 records in BCR since the beginning of the 20^th^ century (Brosens et al. 2013).

Our sampling period occurred only during the spring, from April to June. Ants were located primarily by excavated tumuli and worker activity outside the anthill. Therefore, some species may have been missed if their worker activity is concentrated only underground (i.e. the anthill composed of sand excavated for the nest prospection could not be observed) and their swarming period is later. For example, species belonging to the subgenus Chthonolasius, such as *Lasiusmixtus* (Nylander, 1846) and *Lasiusumbratus* (Nylander, 1846), already recorded in Brussels, are subterranean and their swarming period extends from July to September (Dekoninck et al. 2012, Blatrix et al. 2013). Similarly, the Chao1 index, used to estimate the total number of species present in the study area, is based on empirical data (Chao 1984).

Concerning the species in this study, *L.niger*, is largely dominant. Present in 92% of sites, it seems to appreciate a wide range of conditions for its establishment. Indeed, *L.niger* is considered as an ubiquitous species well known in urban and more widely anthropogenised environments (Wegnez et al. 2012, Blatrix et al. 2013). The other species, much rarer in number, were present in the order of 1-2% each. *L.brunneus, L.flavus, T.caespitum* and *T.impurum* are also characterised as ubiquitous and they are able to establish in urban environments (Wegnez et al. 2012, Blatrix et al. 2013). One of them, *T.caespitum*, is named the "pavement ant" (Kaufmann et al. 2012). Ants of the genus *Tetramorium* particularly like thermophilic sites, whereas *L.brunneus* prefers environments with at least a few trees. Both the *Tetramorium* species have a wide occurence range in the Northern Hemisphere of New and Old World and show euryoecious abilities to live in various habitats, mainly non-forested (Wagner et al. 2017), while *T.caespitum* nests in street medians of New-York City suggesting that this species is able to occupy a particular ecological niche in highly crowded megacities (Pećarević et al. 2010). Except for *L.niger* and *L.flavus*, *L.brunneus*, *L.fuliginosus* and *M.rugulosa* have wide Palearctic distribution according to the global biodiversity information facility (GBIF.org 2023). *L.fuliginosus* and *M.rugulosa* are less frequent in urban environments. The first one is an oligothermal species which prefers environments with tree or forest edges. The second prefers thermophilic and sandy sites. In Wallonia and Brussels, the distribution of the latter species is scattered and rather rare (Wegnez et al. 2012, Blatrix et al. 2013); however, in Flanders, its can be a common species in sandy soils (Dekoninck et al. 2012).

In this study, data were collected in an opportunistic manner. They are described as opportunistic when they were collected without a real standardised protocol and without an experimental design ensuring the geographical representativeness of the sampled sites (van Strien et al. 2013). This method was interesting for an initial investigation of the territory. It allowed a larger area to be explored due to the time saved at each site (Brown and Williams 2019). Indeed, implementing a standardised experimental design with predefined transects would likely have missed some diversity due to low abundance of some species. In addition, the use of participatory science in the study by Noël et al. (2021) revealed spatiotemporal variation in survey effort. Due to the higher abundance of ants under Brussels pavements compared to bees in the previous study, it was decided not to use participatory science and to directly survey as many BCR municipalities as possible to reduce this spatial variation.

### Pavement characterisation

Regarding the composition of the pavements, ants are mostly found under concrete slabs and, to a lesser extent, under natural stones. These proportions can also be explained by the abundance of this type of material used for the construction of Brussels pavements (Centre de Recherches Routières 2012). The nature of the pavement may not be a major criterion for ant colonies. In both cases, these materials have a high thermal conductivity (Laurent and Vuillermoz 1993), allowing for warming of the anthill and brood in early spring (Passera and Aron 2005). About the nature of the joints, most of them were rigid and, therefore, theoretically impermeable, in contrast to the flexible joints made of granular and permeable materials. However, many rigid joints were degraded and no longer constituted a totally impermeable barrier. This was due to the age of the pavement and the vegetation roots that contributed to its degradation. Finally, most of the nests were in the middle of pavements, suggesting that the ants were not bothered by pedestrian traffic.

### Granulometry

The results of the particle size analyses confirm the sandy composition of the pavement subgrades. Indeed, the samples are composed of an average of more than 83% sand. These observations are similar to data collected by Noël et al. (2021) regarding to solitary bee and wasp nests. Sand is a frequently used component of the pavement sub-layer (Centre de Recherches Routières 2012) and can be favourable for a wide variety of Formicidae species including *L.niger* and *T.caespitum* (Wang et al. 2001, Maes et al. 2003, Boulton et al. 2005). It is important to note that the particle size analyses were performed on soil particles excavated by ants and forming a dome above the nest. Therefore, due to the modification of the soil nature caused by ants (Cammeraat and Risch 2008), these analyses may be biased and may not correctly represent the nature of the substrate under the pavements. Indeed, Cammeraat and Risch (2008) showed that, due to the displacement of soil particles by ants, the top of the domes consisted of an accumulation of finer particles than the surrounding soil.

## Conclusions

The ant species observed on the pavements preferentially nest under concrete slab or natural stone with degraded or flexible joints, which are the main materials used for pavements in the BCR. The sand most commonly used for the pavement sub-base is suitable for the establishment of these species. The joints observed are not the most recommended for the comfort of the pedestrian population. Currently, continuous asphalt, concrete pavements or modular concrete pavements with narrow joints are preferred by BCR urban planners to provide sufficient comfort for pedestrians. This study highlights the capacity of urban environments to host biodiversity and, in particular, the pavements considered as refuges for certain species.

## Figures and Tables

**Table 1.** Abundance of sampled species under urban pavements. Identification to species was not possible for two individuals of the genus *Tetramorium* which could be *Tetramoriumcaespitum* (Linnaeus, 1758) or *T.impurum* (Foerster, 1850); that is why the species has not been specified for *Tetramorium*.

**Subfamilies**	**Species**		**Sites**
**Formicinae**	*Lasiusbrunneus* (Latreille, 1798)	2	
	*Lasiusflavus* (Fabricius, 1782)	2	
	*Lasiusfuliginosus* (Latreille, 1798)	1	
	*Lasiusniger* (Linnaeus, 1758)	117	
**Myrmicinae**	*Myrmicarugulosa* Nylander, 1849	1	
	*Tetramoriumcaespitum* (Linnaeus, 1758)	1	
	*Tetramoriumimpurum* (Foerster, 1850)	1	
	*Tetramorium* sp.	2	
